# Role of the IRE1α-XBP1 Axis in IgE-Dependent Activation of Mast Cells

**DOI:** 10.3390/ijms27104532

**Published:** 2026-05-18

**Authors:** Hiroto Kouda, Kazuki Nagata, Riu Saito, Chiharu Nishiyama

**Affiliations:** Department of Biological Science and Technology, Faculty of Advanced Engineering, Tokyo University of Science, 6-3-1 Niijuku, Katsushika-ku, Tokyo 125-8585, Japankazukin@rs.tus.ac.jp (K.N.);

**Keywords:** IgE, IRE1α, mast cell, siRNA, unfolded protein response, XBP1

## Abstract

The IRE1α-XBP1 axis is the most conserved of the three major unfolded protein response (UPR) branches triggered by the endoplasmic reticulum (ER) stress. Although the transcription factor XBP1 is involved in the development and function of several hematopoietic lineages, its role in the activation of mast cells (MCs), which are critical in allergic responses, remains largely unknown. We identified salicylaldehyde, which suppresses IRE1α nuclease activity that is essential for XBP1 production, as an inhibitor of MC activation in our previous screening; therefore, we herein investigated the effects of additional IRE1α inhibitors, 3-methyl-6-bromo-salichylaldehyde (MBSA) and KIRA6, targeting the nuclease domain and kinase domain, respectively, on MC activation. MBSA and KIRA6 suppressed IgE-dependent degranulation of bone marrow-derived MCs (BMMCs) but did not inhibit Ca^2+^ ionophore- or compound48/80-induced degranulation. Treatments with inhibitors of two other branches of UPR, the PERK and ATF6 pathways, did not affect the IgE-induced activation of BMMCs. The intraperitoneal administration of MBSA or KIRA6 significantly suppressed IgE-induced passive anaphylaxis in mice. Furthermore, to examine the effects of XBP1, siRNA-mediated knockdown was performed. The results obtained confirmed that *Xbp1* siRNA introduction reduced the IgE-dependent degranulation of BMMCs in parallel with the knockdown level of *Xbp1* mRNA. Therefore, the IRE1α-XBP1 axis plays a significant role in IgE-dependent and MC-mediated allergic responses and is considered to be a therapeutic target of allergic diseases.

## 1. Introduction

Mast cells (MCs) play important roles in IgE-mediated immune responses, including the pathogenesis of allergic diseases and defenses against parasite infections. In the case of allergic patients carrying hyper IgE, FcεRI expressed on MCs is occupied with antigen-specific IgE, and the cross-linking of multiple IgEs on FcεRI with allergens rapidly activates MCs, resulting in degranulation, eicosanoid release, and cytokine production. In addition, several receptors, such as ST2, MRGPRX2, TLR4, and P2X7, which recognize IL-33, a chemical compound, LPS, and ATP, respectively, are involved in the IgE-independent activation of MCs. Current anti-allergic drugs, such as inhibitors of histamine receptors, leukotriene receptors, and anti-human IgE, target events related to MC activation, demonstrating that the regulation of MC activation is effective for the prevention of allergic diseases [1].

We previously performed screening to identify novel anti-allergic compounds using mouse bone marrow-derived MCs (BMMCs), and selected salicylaldehyde (SA) as the most effective inhibitor of MC activation [2]. The administration of SA suppressed IgE-dependent passive anaphylaxis in mice; however, the molecular mechanisms by which SA inhibit the IgE-dependent activation of MCs in vivo and in vitro remain unclear.

In the present study, we investigated the effects of the IRE1α-XBP1 axis, the most conserved arm of the unfolded protein response (UPR) in MC activation, because it is considered to be a target of SA [3,4,5]. The UPR occurred in response to the endoplasmic reticulum (ER) stress induced by the accumulation of misfolded proteins within the ER lumen. The following events are induced under the UPR condition to maintain cellular homeostasis: the degradation of unfolded proteins is promoted, further protein synthesis is inhibited, and repair mechanisms are facilitated [6]. IRE1α is an enzyme that exhibits both kinase and endoribonuclease activities, and homodimerized and phosphorylated IRE1α creates spliced mature *XBP1* mRNA by cleaving out 26 nucleotides, resulting in the translation of the transcription factor XBP1, which up-regulates the expression of UPR-related genes. XBP1 also regulates several cellular events, such as protein secretion, lipid biosynthesis, glycosylation, and autophagy [6]. XBP1 is involved in various immune system functions, including the differentiation of plasma cells [7], the development and survival of dendritic cells [8] and eosinophils [9], the TLR-mediated cytokine production of macrophages [10], the regulation of type I interferon production in chronically activated pDC [11], and the anti-tumor activity of dendritic cells [12]. On the other hand, the role of XBP1 and/or UPR in MC activation associated with cellular stress remains unclear. Although recent studies examined the role of XBP1 in MC function, the findings obtained were controversial [13,14]. The present study demonstrated a positive role for the IRE1α-XBP1 axis in the IgE-induced activation of MCs.

## 2. Results

### 2.1. Effects of the SA-Related Compound 3-Methyl-6-Bromo-Salichylaldehyde (MBSA) and the XBP1 Splicing Inhibitor KIRA6 on the IgE-Induced Degranulation of BMMCs

**Figure 1:**
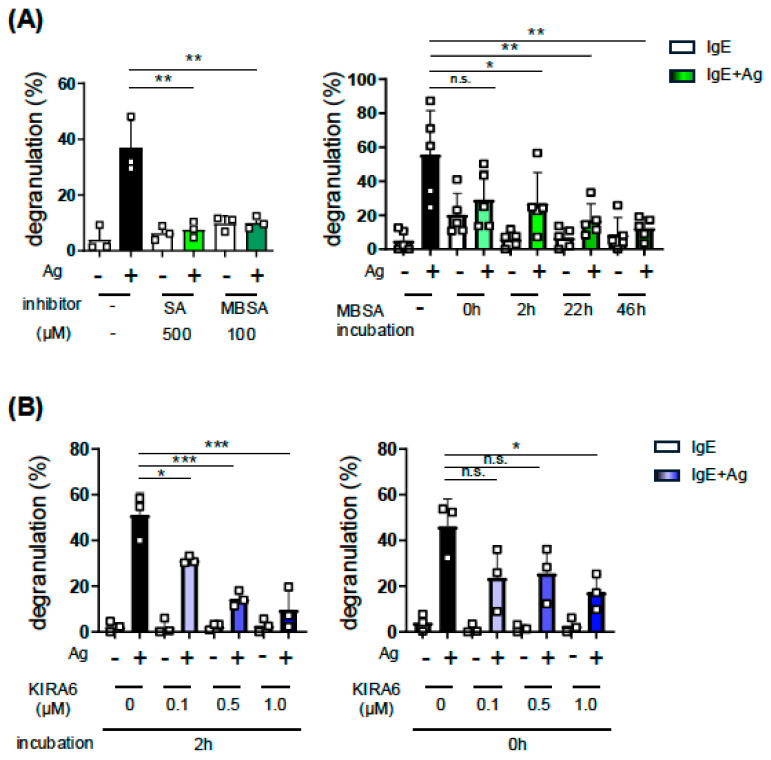
Effects of IRE1α inhibitors, SA, MBSA, and KIRA6, on the IgE-induced degranulation of BMMCs. (**A**) IgE-induced degranulation of BMMCs treated with SA or MBSA (**right**); BMMCs were treated with 500 μM SA or 100 μM MBSA for 48 h, (**left**); BMMCs were treated with 100 μM MBSA for the indicated time. After the treatment with SA or MBSA, BMMCs (5 × 10^5^ cells/mL) were incubated with 200 ng/mL of mouse IgE for 2 h. After washing BMMCs with culture medium twice, BMMCs suspended in Tyrode’s buffer were stimulated with 3 ng/mL of TNP-BSA as an antigen (Ag) for 30 min. Beta-hexosaminidase activities in supernatants and in cells were measured as described in our previous study to assess the extent of degranulation [16]. (**B**) Inhibitory effects of KIRA6 on the IgE-induced degranulation of BMMCs. The indicated concentrations of KIRA6 were added with IgE ((**left**); 2 h before the Ag stimulation) or just when BMMCs were stimulated with Ag (**right**). These experiments were repeated on different days with independently prepared BMMCs from different individuals. The data represent the mean ± SD of independent experiments. Dunnett’s multiple comparison test was used for the statistical analysis between Ag-stimulated BMMCs without an inhibitor (Ctrl) and Ag-stimulated BMMCs with an inhibitor. * *p* < 0.05, ** *p* < 0.01, *** *p* < 0.005, n.s. not significant.

We identified SA as an inhibitor of MC activation through the screening of a chemical/natural compound library in our previous study [2]. Since SA has been used to inhibit *Xbp1* mRNA splicing by IRE1α in keratinocytes [3] and melanoma [4], we herein investigated the role of XBP1 and XBP1-related cellular events, including UPR, in MC activation. We examined the effect of SA-related compound MBSA on MC activation, which exhibits higher inhibitory activity against *XBP1* mRNA splicing than SA [5]. As shown in Figure 1A, left, the treatment with MBSA for 48 h suppressed the IgE-induced degranulation of BMMCs, even at a lower dose than SA, and an inhibitory effect was observed under the condition that MBSA was added to culture media 2 h before or just when the FcεRI-mediated stimulation was induced (Figure 1A, right). We then investigated the involvement of the IRE1α-XBP1 pathway in the IgE-induced activation of MCs by using another inhibitor, KIRA6, which suppresses XBP1 activation by targeting the kinase domain of IRE1α [15], and found that KIRA6 significantly suppressed IgE-induced degranulation (Figure 1B).

Collectively, these results suggest that the inhibition of the IRE1α-XBP1 pathway suppressed the IgE-induced degranulation of MCs.

### 2.2. Effects of MBSA and KIRA6 on IgE-Induced Cytokine Production and IgE-Independent Degranulation of BMMCs

**Figure 2:**
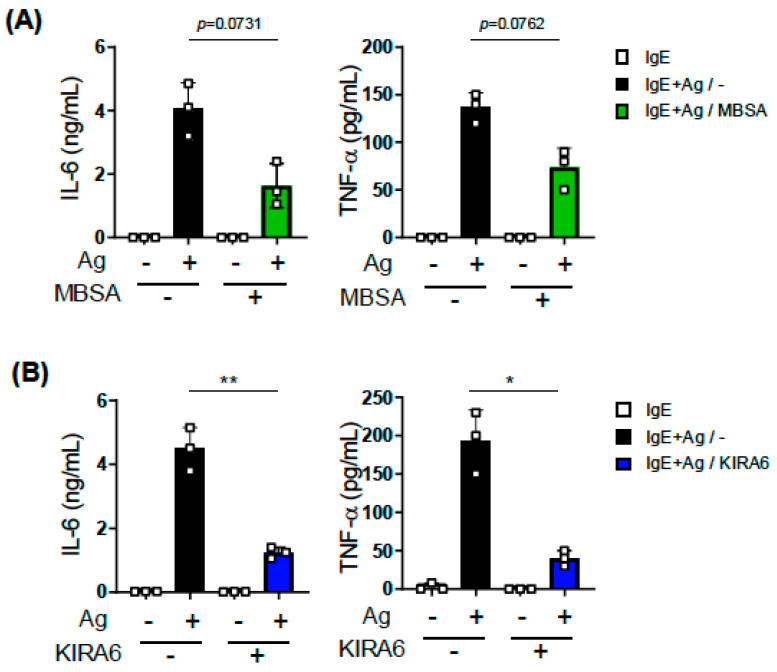
Effects of MBSA and KIRA6 on IgE-induced cytokine secretion from BMMCs. IL-6 (**left**) and TNF-α (**right**) concentrations in the culture supernatants of IgE-stimulated BMMCs with or without the treatment with MBSA (**A**) and KIRA6 (**B**). After a 2 h incubation of BMMCs (5 × 10^5^ cells/mL) with 200 ng/mL of mouse IgE, BMMCs were washed with culture medium and then stimulated with 3 ng/mL of TNP-BSA as an antigen (Ag). Culture supernatants were harvested 3 h after the Ag stimulation for ELISA. MBSA (final concentration 100 μM) was added at 48 h before the Ag stimulation, and KIRA6 (1 μM) was added just before the addition of Ag. These experiments were repeated on different days with independently prepared BMMCs from different individuals. The data represent the mean ± SD of independent experiments. The two-tailed paired Student’s *t*-test was used for the statistical analysis between Ag-stimulated BMMCs without an inhibitor (−) and Ag-stimulated BMMCs with an inhibitor (+). * *p* < 0.05, ** *p* < 0.01.

Upon IgE-dependent activation, MCs cause not only degranulation as the immediate reaction but also release of inflammatory cytokines involved in the late-phase reaction. To examine the effects of XBP1 inhibition on cytokine release, we assessed the amounts of IL-6 and TNF-α produced from IgE-stimulated BMMCs using ELISAs. The results obtained showed that KIRA6 significantly suppressed the IgE-induced secretion of IL-6 and TNF-α from BMMCs (Figure 2B), whereas the effects of MBSA on the release of these cytokines were not significant (Figure 2A).

**Figure 3:**
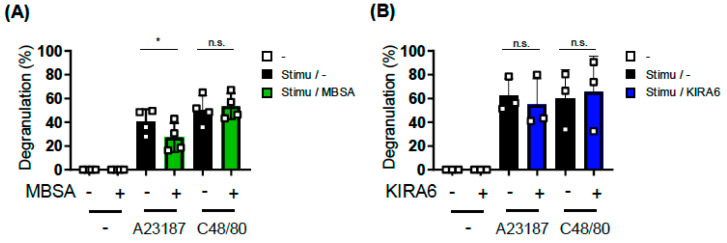
Effects of MBSA and KIRA6 on the Ca^2+^ ionophore- and compound 48/80-induced degranulation of BMMCs. Degranulation levels of BMMCs stimulated by A23187 (**left**) or compound 48/80 (C48/80; **right**) with or without the treatment with MBSA (**A**) and KIRA6 (**B**). BMMCs incubated with 100 μM MBSA for 24 h were stimulated by 1 μM of A23187 (**left**) or 30 μg/mL of C48/80 (**right**) for 30 min (**A**). A total of 1 μM KIRA6 was added just before the addition of A23187 or C48/80 (**B**). Harvested supernatants and collected cells were assessed using the β-hexosaminidase assay (see Materials and Methods). These experiments were repeated on different days with independently prepared BMMCs from different individuals. The data represent the mean ± SD of independent experiments. The two-tailed paired Student’s *t*-test was used for the statistical analysis between without an inhibitor (−) and with an inhibitor (+). * *p* < 0.05, n.s. not significant.

Furthermore, we investigated the effects of inhibitors on the IgE-independent stimulation of BMMCs. Using a b-hexosaminidase assay, we found that compound 48/80-induced degranulation was not affected by the pretreatment with MBSA, whereas Ca^2+^ ionophore-induced degranulation was significantly inhibited by MBSA (Figure 3A). In contrast, KIRA6 did not suppress the IgE-independent degranulation of BMMCs (Figure 3B).

### 2.3. Analysis of the Involvement of Two Other UPR Pathways on MC Activation Using Specific Inhibitors

**Figure 4:**
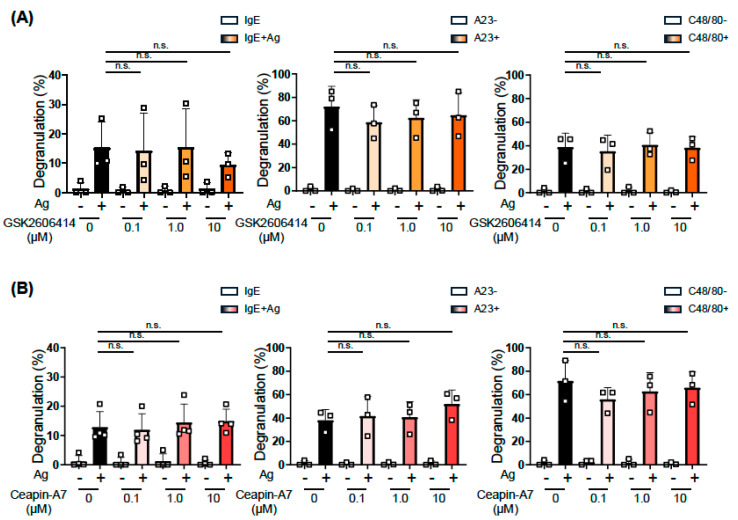
Inhibitors targeting two other pathways did not affect the activation of BMMCs. BMMCs were incubated with the indicated concentrations of GSK2606414, a PERK inhibitor (**A**), or CeapinA-7, an ATF6 inhibitor (**B**), for 22 h before the addition of IgE and for an additional 2 h during the incubation with Ag (**left**), or for 24 h before the addition of A23187 (**center**) and compound 48/80 (**right**). These experiments were repeated on different days with independently prepared BMMCs from different individuals. The data represent the mean ± SD of independent experiments. The two-tailed Dunnett’s multiple comparison test was used for the statistical analysis between without an inhibitor and with an inhibitor. n.s. not significant.

In addition to the IRE1α-XBP1 pathway, the PERK-ATF4 and ATF6 pathways are involved in the UPR. Therefore, we examined the effects of GSK2606414 and CeapinA-7, which inhibit the activation of PERK [17] and the trafficking of ATF6 [18], respectively, on MC activation. We pretreated BMMCs with 0.1–10 μM of GSK2606414 or CeapinA-7 for 24 h based on previously reported experimental conditions. Neither GSK2606414 (Figure 4A) nor CeapinA-7 (Figure 4B) exerted apparent effects on the IgE-, A23187-, or Compound 48/40-induced degranulation of BMMCs.

These results suggest the IRE1α-XBP1-dependent and PERK-ATF4/ATF6-independent manners of the IgE-induced activation of MCs, as observed in other events related to prominent protein synthesis and immuno-responses [7,19,20].

### 2.4. Effects of Tunicamycin on MC Activation

**Figure 5:**
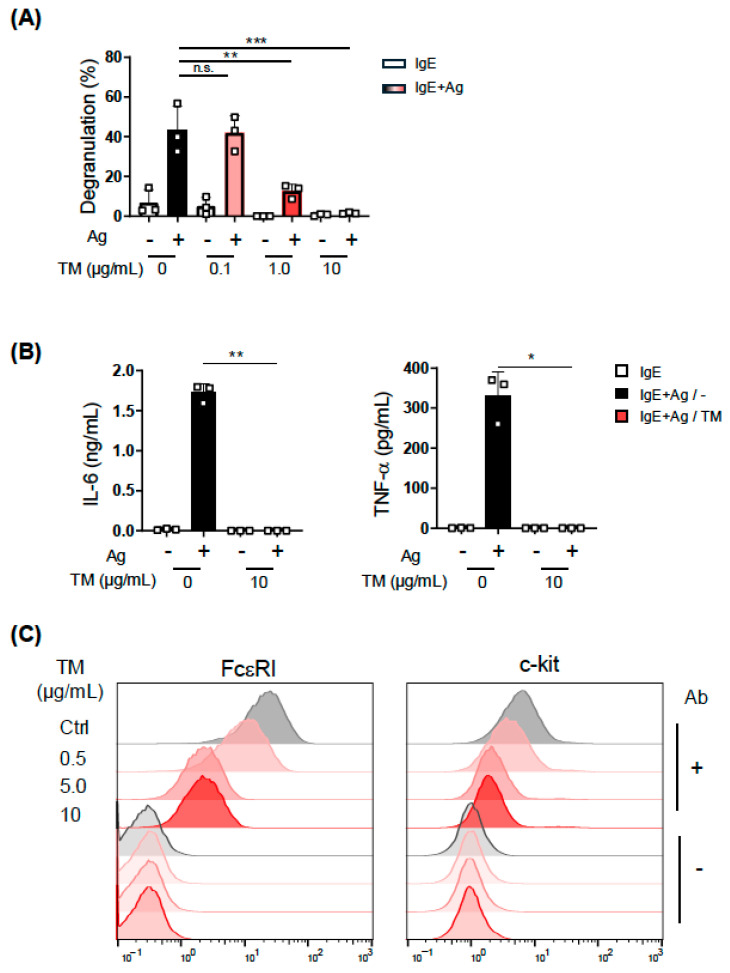
Effects of tunicamycin on MC activation. (**A**) The IgE-induced degranulation of BMMCs treated with tunicamycin. (**B**) IL-6 (**left**) and TNF-α (**right**) production from tunicamycin-treated BMMCs. (**C**) Cell surface expression of FcεRI (**right**) and c-kit (**left**) on tunicamycin-treated BMMCs. BMMCs (5 × 10^5^ cells/mL) were incubated in the presence or absence of the indicated concentration of tunicamycin (TM) for 22 h, and were then subjected to the IgE-induced degranulation assay (**A**), IgE-induced cytokine production (**B**), and to a flow cytometric analysis (**C**). These experiments were repeated on different days with independently prepared BMMCs from different individuals. The data represent the mean ± SD of independent experiments. Dunnett’s multiple comparison test was used in (**A**) and the two-tailed paired Student’s *t*-test was used for the statistical analysis between without TM and with TM (**B**). * *p* < 0.05, ** *p* < 0.01, *** *p* < 0.005, n.s. not significant.

Tunicamycin, which inhibits the *N*-linked glycosylation of proteins, induces the accumulation of unfolded proteins in the ER and subsequent ER stress. In a recent study on the relationship of ER stress with MC degranulation, the intraperitoneal (i.p.) administration of tunicamycin increased the amount of serum Mcpt1 in mice [13], which is a hallmark of MC activation. We then treated BMMCs with tunicamycin to investigate whether the induction of ER stress up-regulates the IgE-induced activation of MCs. As shown in Figure 5A, the tunicamycin pretreatment suppressed the IgE-induced degranulation of BMMCs in a dose-dependent manner. The treatment of BMMCs with 10 μg/mL tunicamycin, which significantly reduced degranulation, completely inhibited the IgE-dependent secretion of IL-6 and TNF-α (Figure 5B). We also revealed that the surface expression of FcεRI on BMMCs was markedly reduced by the treatment with tunicamycin (Figure 5C).

Since *N*-glycosylation is required for multiple processes in MC function, including the transport of FcεRI from the ER to the cell surface [21], these results suggest that tunicamycin did not specifically induce the UPR as predicted in a previous study [13] but inhibited MC activation by instead exerting a broad effect on MCs.

### 2.5. MBSA and KIRA6 Suppressed IgE-Dependent Anaphylaxis in Mice

**Figure 6:**
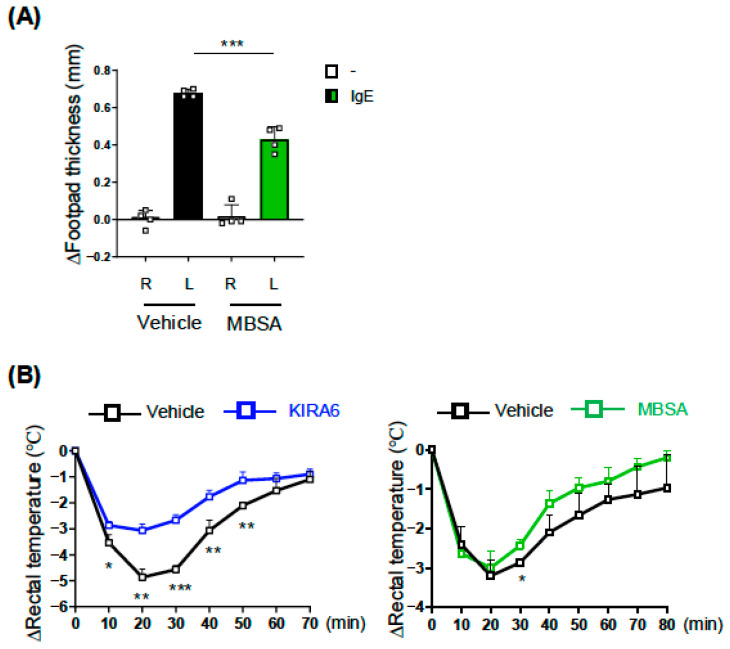
Passive anaphylaxis levels in mice administered MBSA or KIRA6. (**A**) Footpad swelling in passive cutaneous anaphylaxis model mice. Mice were i.p. injected with MBSA (50 mg/kg/day) or 200 μL vehicle (see the Materials and Methods) for 5 days (n = 4 for each group). IgE solution and its vehicle were injected into the left (L) and right (R) footpad, respectively, on day 4, and Ag was i.v. injected on day 5. Footpad thickness 30 min after the Ag injection was measured as described in our previous study [2,22]. The unpaired Student’s *t*-test was used for the statistical analysis between control (Vehicle) and MBSA. *** *p* < 0.005. (**B**) Body temperature of passive systemic anaphylaxis model mice. Mice were i.p. injected with 100 mg/kg/day MBSA or its vehicle (**right**), and 2.5 mg/kg/day KIRA6 (**right**) or its vehicle (**left**) for 3 days (n = 3 for each group). I.v. injections of IgE on day 2 and of Ag on day 3 were performed as previously described [2,22]. The data represent the mean ± SD of independent experiments. The unpaired Student’s *t*-test was used for the statistical analysis between the vehicle and inhibitor. * *p* < 0.05, ** *p* < 0.01, *** *p* < 0.005.

The above-mentioned results of in vitro experiments using BMMCs demonstrated that inhibitors of the IRE1α-XBP1 pathway suppressed the IgE-induced activation of MCs. To examine the effects of these inhibitors on the IgE-dependent allergic response in vivo, we utilized passive anaphylaxis models. As shown in Figure 6A, the i.p. administration of MBSA significantly suppressed ear swelling caused by an i.v. injection of TNP-BSA into IgE-preinjected mice in the passive cutaneous anaphylaxis (PCA) model. We also confirmed that the body temperature reduction induced following the antigen injection in the passive systemic anaphylaxis (PSA) model was attenuated in mice administered MBSA or KIRA6 (Figure 6B).

These results indicate the biological significance of the IRE1α-XBP1 pathway in the IgE-dependent anaphylaxis.

### 2.6. XBP1 Is Involved in IgE-Induced Responses of MCs

**Figure 7:**
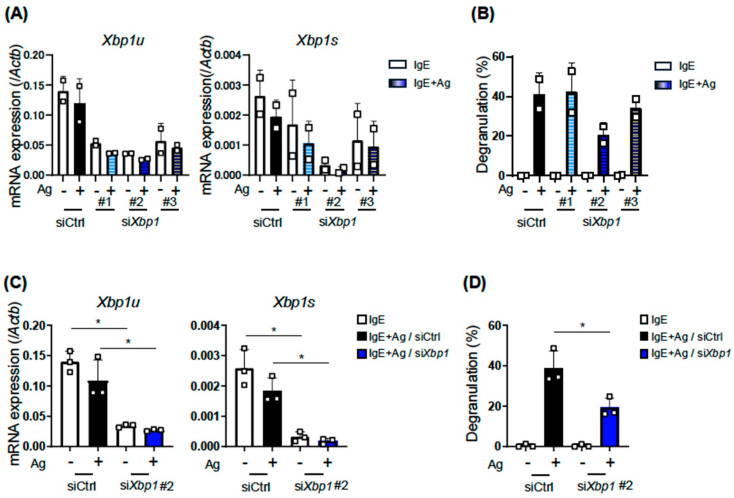
Effects of *Xbp1* siRNA introduction on the IgE-induced activation of BMMCs. (**A**,**C**) mRNA levels of un-spliced *Xbp1* (*Xbp1u*) and spliced *Xbp1* (*Xbp1s*) in siRNA transfected BMMCs, which were harvested 3 h after the Ag stimulation. (**B**,**D**) Degranulation degree of *Xbp1* siRNA-transfected BMMCs. After a 72 h culture from electroporation, BMMCs were divided into two groups: one for mRNA quantification and the other for the degranulation assay. Three different sequences of *Xbp1* siRNAs were used: *Xbp1*-1 (#1, Xbp1-MSS278861), *Xbp1*-2 (#2, Xbp1-MSS278862), and *Xbp1*-3 (#3, Xbp1-MSS278863). These experiments were repeated on different days with independently prepared BMMCs from different individuals. The data represent the mean ± SD of independent experiments. The two-tailed paired Student’s *t*-test was used for the statistical analysis between control siRNA-transfected BMMCs (siCtrl) and *Xbp1* siRNA-transfected BMMCs (si*Xbp1*). * *p* < 0.05.

Two studies on the effects of KIRA6 on MC activation were recently published [14,23]. These studies claimed that KIRA6 suppressed MC activation by inhibiting kinases downstream of FcεRI, such as Lyn and/or Fyn, rather than IRE1α. These conclusions prompted us to investigate the involvement of XBP1 itself in the IgE-induced activation of MCs. To clarify the role of XBP1 in MC activation, we performed a knockdown experiment using siRNA transfection. Among three clones of *Xbp1* siRNAs, *Xbp1*-2 siRNA most effectively reduced the mRNA levels of spliced *Xbp1* (*Xbp1s*), whereas the knockdown efficiency of *Xbp1*-1 and -3 siRNAs was moderate (Figure 7A). A β-hexosaminidase assay on IgE-dependently activated BMMCs revealed that the extent of the degranulation of *Xbp1* siRNA-transfected BMMCs decreased in parallel with the knocked down levels of *Xbp1* mRNA (Figure 7B). We confirmed that the levels of the *Xbp1* mRNA reduction (Figure 7C) and degranulation suppression (Figure 7D) by *Xbp1*-2 siRNA were significant.

Taken together, these results demonstrated that XBP1 was required for the IgE-dependent activation of MCs.

## 3. Discussion

We previously identified SA as the most effective compound that inhibited the IgE-dependent activation of MCs in a screening study aimed at the development of novel anti-allergic drugs [2]. Since SA inhibits the splicing of *Xbp1* mRNA by IRE1α [3,4], which is required for the translation of a functional XBP1 protein, we investigated the involvement of the IRE1α-XBP1 axis in the IgE-dependent activation of MCs in the present study. We revealed that two other IRE1α inhibitors of endoribonuclease and kinase activities, the SA-related compound MBSA [5] and KIRA6 [15], respectively, suppressed the IgE-dependent degranulation of MCs in vitro and IgE-induced passive anaphylaxis in vitro, whereas the inhibitors targeting PERK and ATF6, which are also involved in the UPR as well as IRE1α-XBP1, did not affect the activation of MCs. Furthermore, the knockdown of *Xbp1* mRNA reduced the IgE-dependent degranulation of MCs. Based on these results, we concluded that XBP1 plays a positive role in the IgE-dependent activation of MCs, which may be a therapeutic target of allergic diseases.

XBP1 transactivates the genes related to the secretion of proteins and lipid mediators, including insulin processing protease in the β cells [20], antibody production in plasma cells [7], and prostaglandin synthesis-related enzymes in dendritic cells [24]. Therefore, the genes expressed in activated MCs, which secrete various mediators such as proteases, eicosanoids, and cytokines, may be targets of XBP1. We intended to conduct further detailed analyses of the target genes of XBP1 in MCs.

During the preparation of this manuscript, two studies reporting the anti-allergic effects of KIRA6 were published [14,23]. Wunderle et al. demonstrated that KIRA6 suppressed the Ag-stimulated signaling of MCs by binding to kinases, LYN, FYN, and KIT [23]. The other study claimed that KIRA6 inhibited the kinase activity of Lyn in an IRE1α-independent manner because the knockout of the *Ern1* gene (encoding IRE1α) by genome editing did not affect the Ag-induced activation of RBL-2H3, a rat basophilic cell line [14]. These findings prompted us to perform the knockdown of *Xbp1* in BMMCs in order to reveal the role of XBP1 in the IgE-dependent activation of BMMCs, because an experiment revealing the direct effects of XBP1 is required and primary cells rather than leukemia cells needed to be used. The result showing that the extent of the degranulation of BMMCs decreased in parallel with *Xbp1* mRNA levels indicated a positive role for XBP1 in the activation of MCs. Previous studies on the conditional knockout of *Xbp1* demonstrated the requirement for XBP1 in the development and specific gene expression of hematopoietic cells [8,24,25]. XBP1 maintains the function of immune cells, which is not restricted in the UPR.

A recent study investigated the effects of tunicamycin on MC function [13]. The findings obtained showed that an i.p. injection of tunicamycin for 3 days increased the serum concentration of Mcpt1 in mice without inducing any allergic model, and also that the exposure of BMMCs to tunicamycin for 3 h increased the phosphorylation of kinases, Lyn, Syk, and PLCγ1. Based on these findings, it was concluded that the induction of ER stress by tunicamycin activated MCs. Since the effects of tunicamycin on the IgE-induced activation of MCs was not examined in this study, it is unclear whether the enhanced phosphorylation of kinases leads to the stronger activation of MCs and also which type of stimulation induced Mcpt1 secretion from MCs in tunicamycin-treated mice. However, under our experimental conditions, the tunicamycin treatment markedly suppressed the IgE-dependent activation of BMMCs (Figure 5). This result contrasted with our prediction, based on the previous study, that tunicamycin would enhance the IgE-dependent activation of MCs by inducing ER stress. A marked reduction in cell-surface FcεRI levels in tunicamycin-treated BMMCs is considered to be the primary cause of the responsiveness defect to FcεRI-mediated activation. However, we also recognize that the block of protein glycosylation by tunicamycin broadly affected MC activation-related events because A23187-induced activation was also suppressed in tunicamycin-treated BMMCs.

In the present study, we incubated BMMCs with IRE1α inhibitors for 0~48 h, and found that MBSA exerted stronger inhibitory effects with longer incubation times, whereas KIRA6 added just before the stimulation was the most effective for the suppression of MC activation. This difference may partly reflect the off-target effects of KIRA6 against kinases, Lyn, and/or Fyn [14,23]. In these studies, KIRA6 added 30 min [26] or 1 h [23] before the stimulation inhibited the IgE-dependent activation of MCs. The stronger inhibitory effect of KIRA6 than that of MBSA observed in PSA and cytokine production under our experimental conditions may be due to the additional target sites of KIRA6. However, we do not exclude the role of KIRA6 against IRE1α because *Xbp1s* mRNA levels in Ag-stimulated BMMCs were generally reduced in the presence of 1 mM KIRA6 [23]. Our preliminary experiment showed that mRNA levels of *Xbp1s* in BMMCs decreased 2 to 48 h after MBSA treatment; however, further experiments are needed to clarify how *Xbp1s* mRNA levels change over time following KIRA6 treatment under our experimental conditions. To evaluate the IRE1α-XBP1-dependency of the inhibitory effects of MBSA and KIRA6, experiments could be conducted using MCs lacking XBP1. This point could potentially be resolved by performing a degranulation assay of BMMCs generated from *Xbp1*-deficient mice to determine whether MBSA and KIRA6 exert additional inhibitory effects on *Xbp1-*knockout BMMCs. Furthermore, the use of *Xbp1-*knockout MCs may demonstrate that XBP1 is involved in the inhibitory effect of MBSA on A23187-induced MC activation.

The involvement of the IRE1α-XBP1 axis in the IgE-dependent activation of MCs has been controversial: an IRE1α deficiency did not affect the IgE-induced activation of RBL-2H3 cells [14], whereas IgE-dependent Syk phosphorylation and calcium mobilization were completely inhibited in *Xbp1* knockout mouse MCs [13]. The results obtained using IRE1α inhibitors and *Xbp1*-targeting siRNA suggest that this pathway plays a positive role in IgE-dependent activation; however, further studies are needed to resolve this issue. A previous study using *Ern1*-knockout BM dendritic cells have suggested that IRE1α-XBP1 signaling plays an essential role in prostaglandin biosynthesis [24]. Furthermore, we have demonstrated that prostanoids, particularly PGE_2_, suppressed IgE-dependent activation of MCs [22,27]. Recently, we observed increased levels of *Ptgs* mRNAs in MBSA-treated or *Xbp1* siRNA-transfected BMMCs. It may be interesting to evaluate the involvement of XBP1 in PG-related genes in MCs. We are currently attempting to identify the target gene(s) of XBP1 in MCs, which we expect to provide insights into the role of XBP1 in MC activation.

Finally, it is important to note that because XBP1 is involved in multiple processes across various organs and cells, it is necessary to consider the potential side effects that may arise from systemic administration of the inhibitors of XBP1. Identifying the target gene(s) of XBP1 in MCs could lead to development of specific treatments for allergic diseases.

## 4. Materials and Methods

### 4.1. Mice and Cells

C57BL/6J male and female mice (Japan SLC, Hamamatsu, Japan) housed under specific pathogen-free conditions were utilized as a passive anaphylaxis model (6–8 weeks old) or were sacrificed to generate BMMCs (6–10 weeks old). BMMCs were prepared under recombinant mouse IL-3 (BioLegend, San Diego, CA, USA) supplemented conditions as previously described [28]. Mice were maintained under specific pathogen-free-conditions, and all experiments using mice were performed following the guidelines of the Institutional Review Board of Tokyo University of Science. The present study was approved by the Animal Care and Use Committees of Tokyo University of Science: K22005.

### 4.2. Reagents

Anti-TNP mouse IgE (clone IgE-3, BD Bioscience, San Jose, CA, USA), TNP-BSA (LSL, Tokyo, Japan), 4-nitrophenyl-*N*-acetyl-β-D-glucosaminide (#N0866, Tokyo Chemical Industry, Tokyo, Japan), SA (#S0004, Tokyo Chemical Industry), MBSA (#B1487, Tokyo Chemical Industry), KIRA6 (#S8658, Selleck Chemicals, Houston, TX, USA), GSK2606414 (#T2614, TargetMol, Boston, MA, USA), Ceapin-A7 (#T9110, TargetMol), A23187 (#11016, Funakoshi, Tokyo, Japan), compound 48/80 (#C2313, Sigma-Aldrich, St. Louis, MO, USA), tunicamycin (#ab120296, Abcam, Cambridge, UK), and DMSO (07-4860-5, Sigma-Aldrich) were purchased from the indicated sources.

### 4.3. Degranulation of MCs

A degranulation assay was performed using a previously described method [16] by sensitizing BMMCs (5.0 × 10^5^) with 0.2 μg/mL anti-TNP mouse IgE in 1 mL medium at 37 °C for 2 h, and then stimulating these cells with 3 ng/mL TNP-BSA in 200 μL Tyrode’s buffer after washing with Tyrode’s buffer. After a 30 min stimulation, culture supernatants were harvested for the β-hexosaminidase assay [16].

### 4.4. Quantification of mRNAs

RNAzol RT Reagent (#RN190, Cosmo Bio, Tokyo, Japan) and ReverTraAce qPCR RT Master Mix (#FSQ-201, TOYOBO, Osaka, Japan) were used for the extraction of total RNA and for the synthesis of cDNA, respectively. Quantitative real-time PCR was performed on a StepOne Real-Time PCR System (Applied Biosystems, Waltham, MA, USA) with THUNDERBIRD SYBR qPCR Mix (#QPS201, TOYOBO) using the following primers.

*Xbp1*u-F; 5′-GACAGAGAGTCAAACTAACGTGG-3′

*Xbp1*u-R; 5′-GTCCAGCAGGCAAGAAGGT-3′

*Xbp1*s-F; 5′-AAGAACACGCTTGGGAATGG-3′

*Xbp1*s-R; 5′-CTGCACCTGCTGCGGAC-3′

*Actb*-F; 5′-AGATGACCCAGATCATGTTTGAGA-3′

*Actb*-R; 5′-CACAGCCTGGATGGCTACGTA-3′

### 4.5. ELISA

The concentrations of mouse IL-6 and TNF-α were assessed using ELISA MAX Standard/Deluxe Set-series ELISA kits (#431316 for IL-6; #430901 and #430916 for TNF-α).

### 4.6. Flow Cytometric Analysis

BMMCs pretreated with 1 mg/mL Fc block (clone 93, BioLegend) were stained with 2.5 mg/mL DAPI (#11034-56, Nacalai Tesque, Kyoto, Japan), PE anti-mouse FceRI (clone MAR1, BioLegend, 1:500), FITC anti-mouse c-kit (clone 2B, BioLegend, 1:200), and APC anti-mouse c-kit (clone ACK2, BioLegend, 1:200). Fluorescence was detected by a FACSLyric Analyzer (BD Pharmingen, San Diego, CA, USA) or a MACS Quant Analyzer (Miltenyi Biotec, Tubingen, Germany), and data were processed with FlowJo software v10.10.0 (Tomy Digital Biology, Tokyo, Japan).

### 4.7. Transfection of siRNA

Small interfering RNAs for mouse *Xbp1*; *Xbp1*-1 (Xbp1-MSS278861), *Xbp1*-2 (Xbp1-MSS278862), *Xbp1*-3 (Xbp1-MSS278863), and Stealth RNAi siRNA Negative Control MedGC (#12935-300) were purchased from Invitrogen (Carlsbad, CA, USA). BMMCs (2.0–6.0 × 10^6^) were transfected with 10 mL of 20 mmol/L siRNA using the Neon transfection system (Invitrogen) as previously described [28].

### 4.8. Passive Anaphylaxis

PSA and PCA were introduced into C57BL/6 mice by the administration of anti-TNP mouse IgE and TNP-BSA as described in our previous study [22]. MBSA (50–100 mg/kg), KIRA6 (2.5 mg/kg), or vehicle (saline:PEG:Tween-80:DMSO = 10:8:1:1 for MBSA, saline:DMSO:Tween-80 = 18:1:1 for KIRA6) was administered via an i.p. injection once per day.

### 4.9. Statistical Analysis

A two-tailed Student’s *t*-test was used to compare two samples. To compare more than three samples, a one-way ANOVA followed by Dunnett’s multiple comparison test was performed. *p*-values < 0.05 were considered to be significant.

## Data Availability

The raw data supporting the conclusions of this article will be made available by the authors, without undue reservation.

## Abbreviations

The following abbreviations are used in this manuscript:

BMMC	bone marrow-derived mast cell
ER	endoplasmic reticulum
MBSA	3-methyl-6-bromo-salichylaldehyde
MC	Mast cell
SA	salicylaldehyde
UPR	unfolded protein response
